# Can “Port-Wine Marks” on the Face Be Cured?—Yes

**Published:** 1876-10

**Authors:** Balmanno Squire

**Affiliations:** Surgeon to the British Hospital for Diseases of the Skin, London


### CAN "PORT-WINE MARKS" ON THE FACE BE CURED? YES.

By BALMANNO SQUIRE, M.D., Surgeon to the British Hospital for Diseases of the Skin, London.

Few lesions of the skin are more hideously disfiguring than the congenital "Port-wine mark" of the face. I refer to the flat vascular nævus which may so often be met with in every country, causing the greater part (often) of one side of the face to present a livid, dark crimson color, and conferring an almost demonical appearance of the unfortunate subject of this forbidding deformity. So many adults of all classes of society may be seen going about with this lesion in its pristine condition, that it is clear at once that nothing is commonly contrived for its re-relief, and a little experience suffices to prove that any attempt at interference with this deformity is commonly regarded by the profession with disfavor. By some, the possibly uncontrollable hemorrhage is the fear entertained; by others, ths scar that would ensue from the only means that seems to be free from the objection cited—cauterization—is properly a reason for refraining. However, as I have satisfactorily ascertained, the disfigurment can be removed without leaving any trace of its former existence, or of the means employed for its removal, and that by a very simple, safe, painless, speedy and easy procedure.

For the purpose in view I employ a cataract needle, the head of which is about four times the size of the ordinary cataract needle. With this needle I scarify the affected skin, making cleanly cut and parallel incisions over the affected area, and even also a little beyond it. The incisions are spaced apart one-sixteenth of an inch. In order to render the operation painless, and at the same time prevent any flow of blood interfering with the draughtmanship of the lines, I first freeze the skin thoroughly by means of Dr. Richardson's æther spray apparatus. Having performed the operation over a limited area, I press on the scarified portion of skin with the fingers for about ten minutes, gently but firmly. At the end of this time all bleeding has definitely ceased. During the pressure a piece of white blotting paper is interposed between the fingers and the skin. The only styptic I employ is that of pressure employed as that above described. As to the depths of the incisions, they should be made of such depth as nearly to divide the entire thickness of the cutis vera. Within a fortnight, if deftly performed, the operation has done its work without leaving trace of any kind save a notable and most gratifying improvement. No scars are left by it. However a precaution needs to be stated. No lateral traction must be made on the scarified skin either during or within half an hour after the performance of the operation. In exercising styptic pressure after the operation, this essential precaution must be kept in view. When, in any case, any traction has been accidently made on the skin in a direction transverse to the direction of the cuts, they gape slightly in consequence. The gaping cuts become plugged with wedged shaped clots, and, as an invariable fact, indelible linear scars are thus produced. If traction be avoided, no trace is left of the operation. Sometimes one operation alone will not suffice, a second or even a third may be required. In such cases the direction followed by the linear incisions of the first operation should be carefully remembered, and at the second operation the parallel linear cuts should be made to cross obliquely the direction of the original cuts, say at an angle of forty-five degrees. If a third operation be needed, the cuts should again follow a different direction, that is to say, they should cross the direction of the original cuts at right angels.

After the operation any exudition of clot or scab should be washed off carefully the next day by a soft camel's hair brush and cold soap and water, followed by a soft piece of sponge wet with cold water only.

The operation conducted as above is absolutely painless. Very slight temporary swelling follows it. No permanent trace is left by it. It does its work finally within a fortnight. No hemorrhage accompanies it, nor is it attended by risk of any kind. It offers to a number of hideously deformed persons an escape from their misfortune which may be safely recommended, and confidently offered by any practitioner. The results obtained by it are at once gratifying to the practitioner and satisfactory to the patient.—*Archives of Dermatology.—Cin. Lancet and Observer.*

